# Supplementary material for: Viscoelastic Niches Shape γδ T-Cell Phenotype and Effector Function
Source: bioRxiv. 2026 Jul 24:2026.07.23.740350. Preprint. [Version 1] doi: 10.64898/2026.07.23.740350 (PMC13419839; doi:10.64898/2026.07.23.740350)
Supplement: Supplement 1 [file media-1.pdf]

Viscoelastic Niches Shape γδ T-Cell Phenotype and Effector Function

Supplementary Figures and Tables

Favour Omafuvwe Obuseh [1,3], Michelle Chang [2], Joshua Price [2,3], Tania To [2,3], Kyle Ruark[2,3],Leah Lourenco[2], Bogdan Budnik[3], David Mooney[2,3]

Affiliations:

1. Harvard-MIT Program in Health Sciences and Technology (HST), Cambridge, MA, USA
2. School of Engineering and Applied Sciences (SEAS), Harvard University, Cambridge, MA, USA
3. Wyss Institute for Biologically Inspired Engineering, Harvard University, Boston, MA, USA

Supplementary Figures

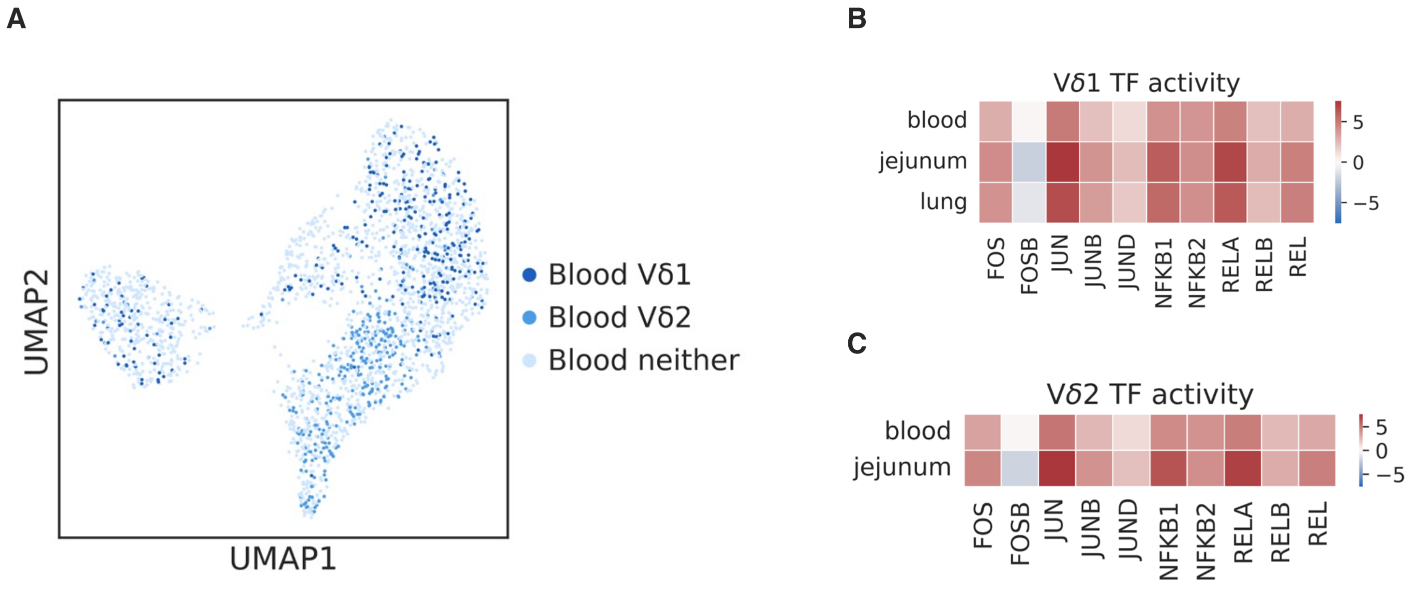

Figure S1. **Single cell RNA-sequencing reveals tissue-dependent transcriptional states in primary human γδ T-cells**
(A) UMAP of γδ T-cells from blood, indicating Vδ1 and Vδ2 identities (sequencing data from Hao et al., 2021). (B) CollecTRI ULM t-values for FOS, JUN, and NFKB family transcription factors in blood, jejunum, and lung Vδ1 cells; mean across donors. (C) CollecTRI ULM t-values for FOS, JUN, and NFKB family transcription factors in blood and jejunum Vδ2 cells; mean across donors. B represent 4 jejunum, 2 lung, and 6 blood donors, C represent 4 jejunum, and 6 blood donors

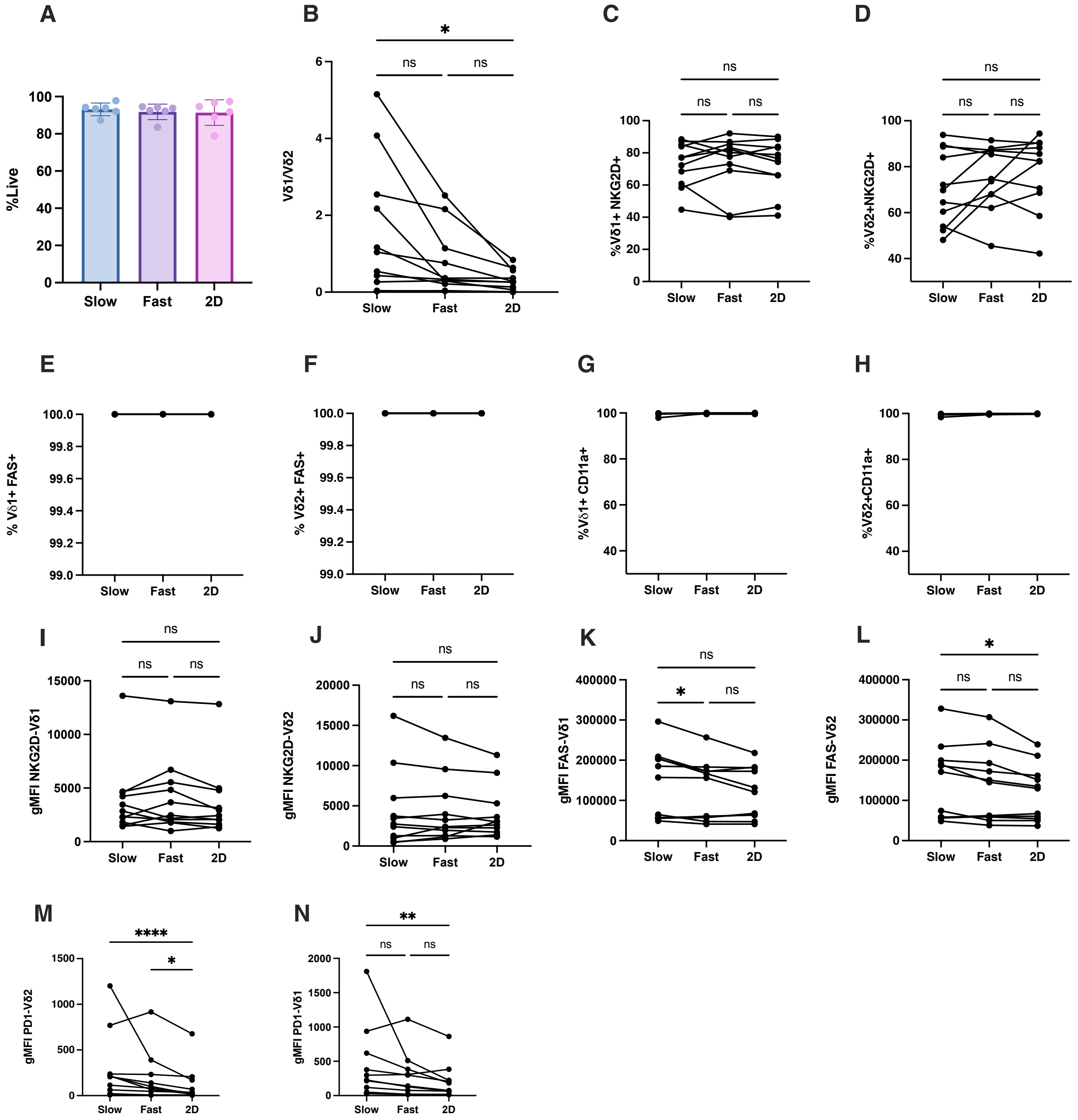

Figure S2. Viscoelasticity Effect on Activation

(A) Percentage of viable γδ T-cells after encapsulation in slow-relaxing, fast-relaxing, or unencapsulated (2D) conditions. (B) Vδ1/Vδ2 ratio after encapsulation in slow-relaxing, fast-relaxing, or unencapsulated (2D) conditions.(C) Percentage of Vδ1^{+} NKG2D^{+} cells after encapsulation in slow-relaxing, fast-relaxing, or unencapsulated (2D) conditions.(D) Percentage of Vδ2^{+} NKG2D^{+} cells after encapsulation in slow-relaxing, fast-relaxing, or unencapsulated (2D) conditions.(E) Percentage of Vδ1^{+} FAS^{+} cells after encapsulation in slow-relaxing, fast-relaxing, or unencapsulated (2D) conditions.(F) Percentage of Vδ2^{+} FAS^{+} cells after encapsulation in slow-relaxing, fast-relaxing, or unencapsulated (2D) conditions.(G) Percentage of CD11a^{+} Vδ1 cells after encapsulation in slow-relaxing, fast-relaxing, or unencapsulated (2D) conditions.(H) Percentage of CD11a^{+} Vδ2 cells after encapsulation in slow-relaxing, fast-relaxing, or unencapsulated (2D) conditions.(I) Geometric mean fluorescence intensity (gMFI) of NKG2D in Vδ1^{+} cells after encapsulation in slow-relaxing, fast-relaxing, or unencapsulated (2D) conditions.(J) Geometric mean fluorescence intensity (gMFI) of NKG2D in Vδ2^{+} cells after encapsulation in slow-relaxing, fast-relaxing, or unencapsulated (2D) conditions.(K) Geometric mean fluorescence intensity (gMFI) of FAS in Vδ1^{+} cells after encapsulation in slow-relaxing, fast-relaxing, or unencapsulated (2D) conditions.(L) Geometric mean fluorescence intensity (gMFI) of FAS in Vδ2^{+} cells after encapsulation in slow-relaxing, fast-relaxing, or unencapsulated (2D) conditions.(M) Geometric mean fluorescence intensity (gMFI) of PD-1 in Vδ1^{+} cells after encapsulation in slow-relaxing, fast-relaxing, or unencapsulated (2D) conditions.(N) Geometric mean fluorescence intensity (gMFI) of PD-1 in Vδ2^{+} cells after encapsulation in slow-relaxing, fast-relaxing, or unencapsulated (2D) conditions. For panel A, n = 6 donors. For panel B, n = 11 donors. For panels C–N, n = 11 donors. Bar graphs show mean ± s.d. Statistical significance was determined using one-way ANOVA with Tukey's multiple comparisons test. *$P < 0.05$, **$P < 0.01$, ***$P < 0.001$, ****$P < 0.0001$; ns, not significant.

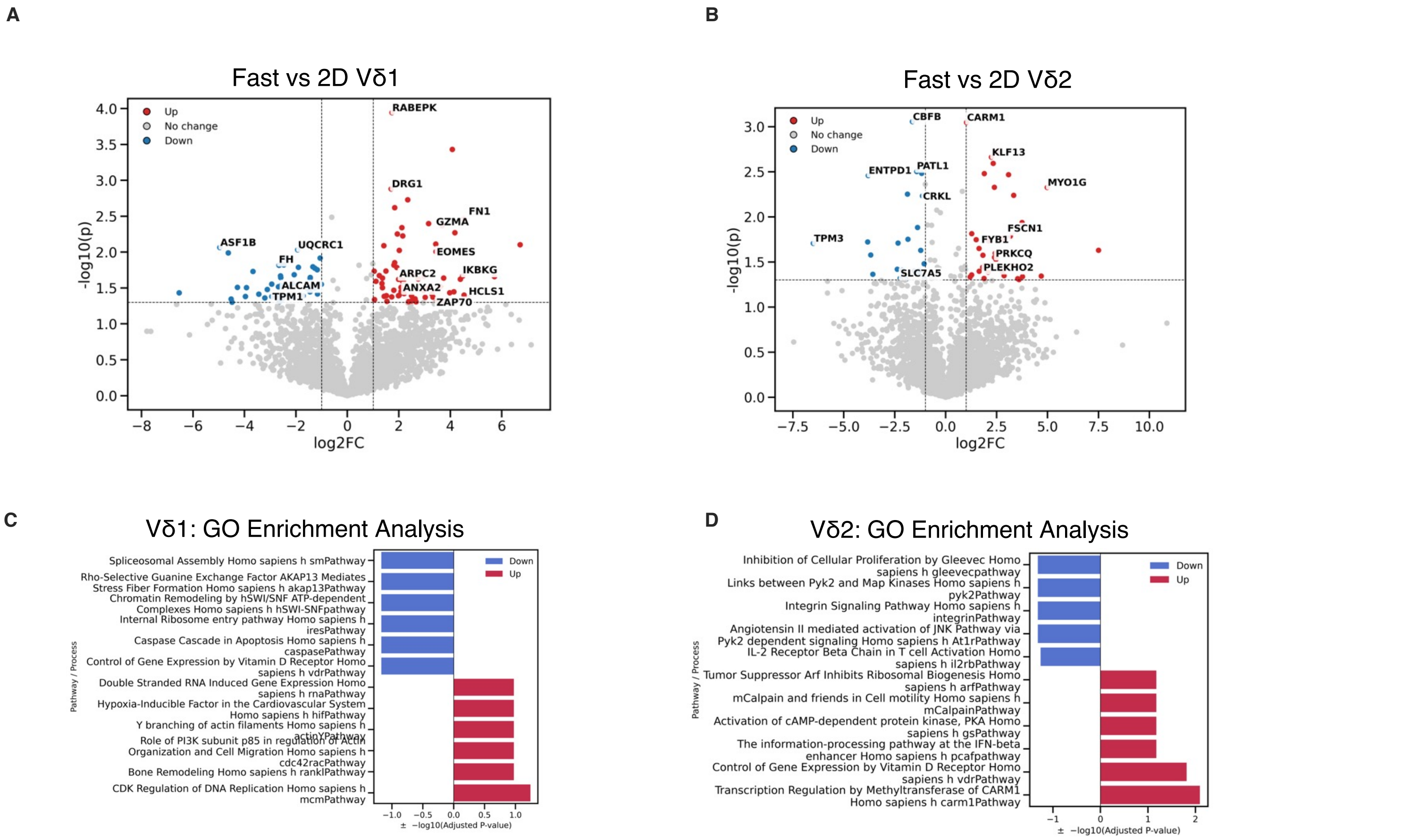

Figure S3. Encapsulation Drives Proteomic Remodeling in γδ T-cells

(A) Volcano plot comparing FACS-sorted Vδ1 cells cultured in fast-relaxing matrix conditions versus non-encapsulated. (B) Volcano plot comparing FACS-sorted Vδ2 cells cultured in fast-relaxing matrix conditions versus non-encapsulated conditions. (C) Enrichr over-representation analysis (ORA) of Gene Ontology (GO) biological processes (BioCarta 2016) for Vδ1 cells cultured in fast-relaxing matrix conditions versus non-encapsulated conditions. (D) Enrichr over-representation analysis (ORA) of Gene Ontology (GO) biological processes (BioCarta 2016) for Vδ2 cells cultured in fast-relaxing matrix conditions versus non-encapsulated conditions. For panels A,C, n = 4 donors; for panels B,D, n = 3 donors. Statistical significance in A,B, was determined using a paired t-test *$P < 0.05$, **$P < 0.01$, ***$P < 0.001$; ns, not significant.

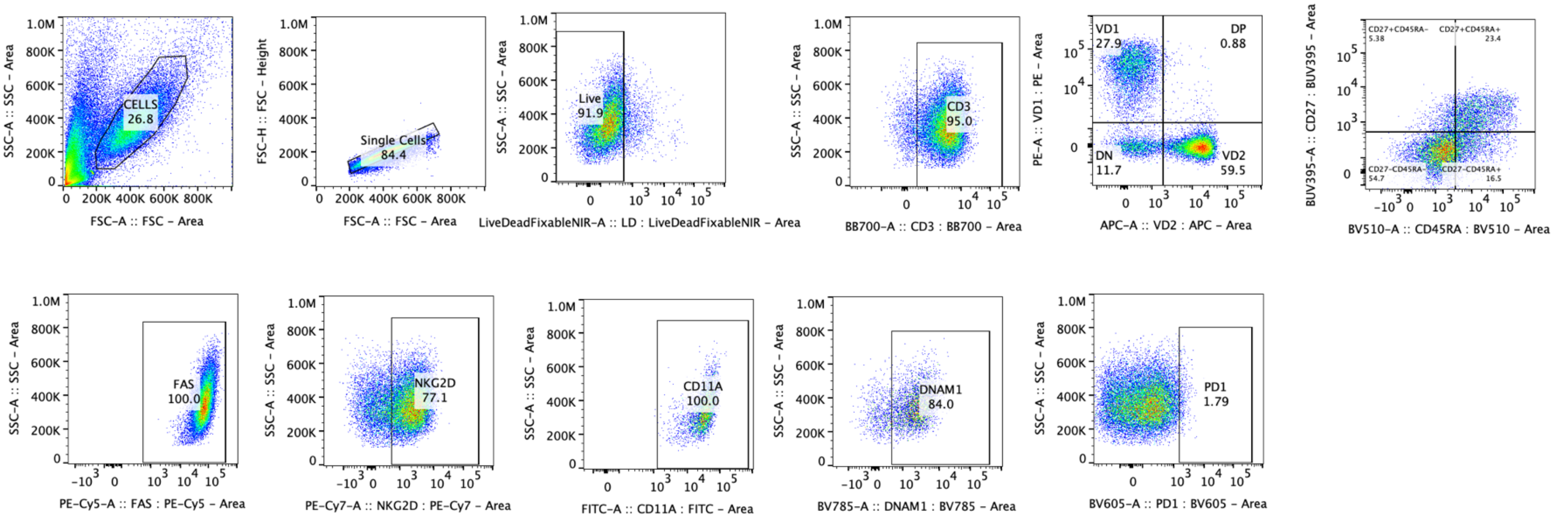

Figure S4. Gating Strategy

Gating performed using fluorescence minus one (FMO) control.

Staining Antibody	Manufacturer	Cat#
CD3 (RB705)	BD Biosciences	570237
TCRγδ (PE/Dazzle)	BioLegend	331226
TCR Vδ1 (PE)	Thermo Fisher	12-5679-42
TCR Vδ2 (APC)	BioLegend	331418
CD27 (BUV395)	Thermo Fisher	363027942
CD45RA (Brilliant Violet 510)	BioLegend	304142
PD-1 (BV605)	BioLegend	367426
NKG2D (PE-Cy7)	Thermo Fisher	25-5878-42
CD11a (FITC)	Thermo Fisher	11-0119-42
DNAM-1 (BV785)	BioLegend	338322
NKp30 (BV711)	BioLegend	325218
CD95/FAS (PE-Cy5)	Thermo Fisher	15095942
Live/Dead NIR viability dye	Thermo Fisher	L10119
TIGIT (BV421)	BioLegend	372710
IFNγ (FITC)	BD Biosciences	554551
Granzyme B (PE-Texas Red)	Invitrogen	GRB17
TNFα (BV421)	Thermo Fisher	404-7349-42

Table S1. Antibodies used for experiments

List of antibodies used for flow cytometry, with manufacture’s catalog number provided. The antibodies were used at 1:60 dilution, except for Live Dead NIR which was 1:1000 dilution.
